# Draft Genome Sequence of the Plant Growth-Promoting *Streptomyces* sp. Strain 6-11-2

**DOI:** 10.1128/MRA.00877-19

**Published:** 2019-11-21

**Authors:** Ayman Ztouti, Natsumi Saito, Junna Sasaki, Takashi Yamaguchi, Nobuo Araki, Mamoru Oshiki

**Affiliations:** a Department of Civil Engineering, National Institute of Technology, Nagaoka College, Nagaoka, Niigata, Japan; b Department of Creative Engineering, National Institute of Technology, Tsuruoka College, Tsuruoka, Yamagata, Japan; c Department of Chemical and Biological Engineering, National Institute of Technology, Tsuruoka College, Tsuruoka, Yamagata, Japan; d Department of Science of Technology Innovation, Nagaoka University of Technology, Nagaoka, Niigata, Japan; University of Maryland School of Medicine

## Abstract

A genome sequence of *Streptomyces* sp. strain 6-2-11, a bacterium capable of promoting plant growth, was determined using the Illumina NovaSeq and MinION sequencers. An 8.9-Mb genome sequence composed of 3 contigs was successfully assembled, where 7,980 gene-coding regions, 6 *rrn* operons, and 86 tRNA genes were located.

## ANNOUNCEMENT

**FIG 1:**
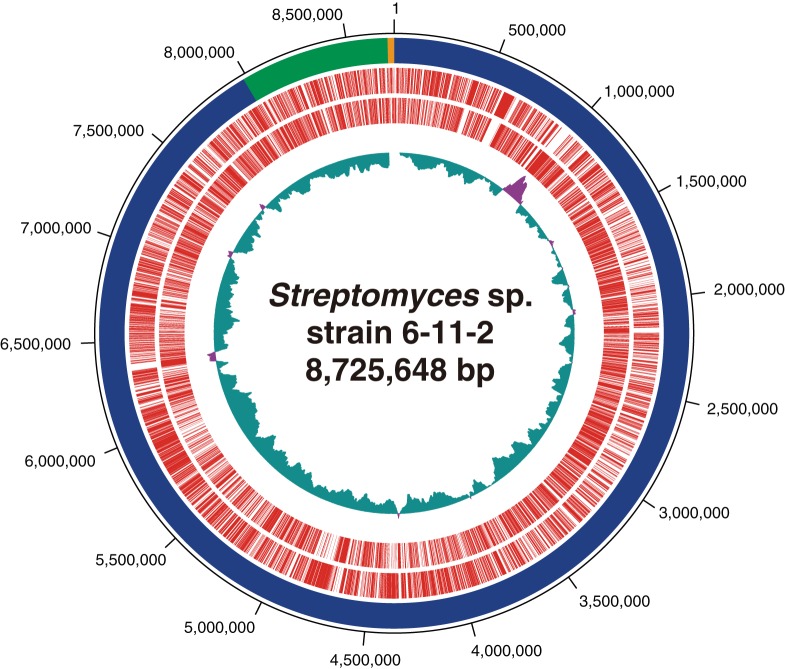
Circular map of the *Streptomyces* sp. strain 6-11-2 genome; the outermost circle represents contig 1 (blue), contig 2 (green), and contig 3 (yellow). The second and third outermost circles represent forward and reverse coding DNA sequences (CDSs), respectively, shown in red. The innermost circle represents the G+C skew. Green and magenta represent regions lower and higher than the center (0.05), respectively.

*Streptomyces* spp. are Gram-positive bacteria affiliated with the bacterial phylum *Actinobacteria* and are well recognized as producers that synthesize a variety of secondary metabolites, including antibiotics. We have recently isolated a *Streptomyces* sp. strain, 6-11-2, capable of promoting plant (i.e., Arabidopsis thaliana) growth from the surface soil of a cherry blossom tree (0- to 5-cm depth near the tree) using a humic acid-vitamin (HV) agar plate containing nalidixic acid and cycloheximide as the antibiotics. *Streptomyces* sp. strain 6-11-2 was obtained after a 1-week incubation on the HV agar at 27°C. Thus far, the gene set involved in the promotion of plant growth by *Streptomyces* sp. strain 6-11-2 had never been characterized genetically and biochemically. Here, we report the draft genome sequence of *Streptomyces* sp. strain 6-11-2. For DNA extraction, the freeze-stored spore suspension was inoculated into GYM broth (glucose, yeast extract, malt extract, and trace elements). After 2 days of incubation at 27°C with shaking at 180 rpm, cells were collected by centrifugation and washed with phosphate-buffered saline. Cells were lysed using proteinase K and SDS, and genomic DNA was purified using a silica membrane spin column supplied as part of the DNeasy blood and tissue kit (Qiagen K.K., Tokyo, Japan). For the Illumina sequencing, the total genomic DNA was sheared into 350-bp fragments using an E200 ultrasonicator (Covaris, Woburn, MA, USA). The genomic DNA was subjected to 150-bp paired-end Illumina sequencing and Nanopore sequencing using the Illumina NovaSeq (TruSeq Nano DNA kit) and MinION (flow cell version R9 and rapid sequencing kit) sequencers, respectively. Default parameters were used for all software, unless otherwise specified. The Illumina and Nanopore sequencing produced 8,466,332 (1,277,897,715 bp of total nucleotides) and 80,460 (272,682,559 bp) sequence reads, respectively. Only the reads stored in the “pass” folder were used in the assembly. Trimming and filtering of the Illumina and MinION reads were performed using the Trimmomatic v0.36 (Phred quality score, ≤30) (1) and Nanofilt v2.3.0 (Phred quality score, ≤8; 50 bp of the 5′ end) (2) software programs, respectively, and the reads were assembled into a 8.9-Mb genome sequence composed of 3 contigs (G+C content, 71.3%; >177× coverage) (Fig. 1) using the Unicycler v0.4.1 software (3). Gene prediction and annotation were performed using the DFAST annotation pipeline (4), and 7,980 gene-coding regions, 6 *rrn* operons, and 86 tRNA genes were found. The quality of the assembled genome sequence was evaluated using a benchmarking universal single-copy ortholog (BUSCO) software v3 (5) and the *Actinobacteria* odb9 data set. All of the 357 BUSCOs were located in the assembled genome. The full-length sequence of the *Streptomyces* sp. strain 6-11-2 16S rRNA gene shared 99.1% similarity with that of Streptomyces niveoruber strain MA1 (GenBank accession number JF827351), and we tentatively designated our isolate *Streptomyces* sp. strain 6-11-2. A gene set responsible for the biosynthesis of secondary metabolites was screened using the antiSMASH software v5.0 (6). The 23 candidate gene clusters involved in the biosynthesis of secondary metabolites (e.g., bacteriocin, terpene, siderophore, and polyketide) were found from the *Streptomyces* sp. strain 6-11-2 genome. Further studies need to investigate how *Streptomyces* sp. strain 6-11-2 promotes plant growth and to identify a gene set responsible for this metabolism.

### Data availability.

The *Streptomyces* sp. 6-11-2 genome sequence was deposited into the DDBJ nucleic acid sequence database under the accession numbers BJOR01000001, BJOR01000002, and BJOR01000003. The Illumina and Nanopore reads are available in the DDBJ Sequence Read Archive (DRA) under accession number DRA008603. The fast5 files obtained by Nanopore sequencing are also available in the DDBJ DRA under accession number DRZ014498.
